# Treatment decisions and surgery variables are predictors of better physical function after total hip and knee arthroplasty: a retrospective cohort study

**DOI:** 10.1186/s42836-025-00313-2

**Published:** 2025-06-04

**Authors:** Janis Nikkhah, Lukas Schöner, Carlos J. Marques, Christoph M. Pros, Reinhard Busse

**Affiliations:** 1 Department of Health Care Management, Technical University Berlin, 10623 Berlin, Germany; 2 Department of Performance, Neuroscience, Therapy, and Health, Institute of Interdisciplinary Exercise Science and Sports Medicine, Medical School Hamburg (University of Applied Sciences and Medical University), Hamburg, 20457 Germany

**Keywords:** Patient-reported outcome, Value-based health care, Quality of care, Treatment decisions, Surgery team composition

## Abstract

**Background:**

Demographic factors are driving the further increase of total hip (THA) and total knee arthroplasty (TKA) volumes in the next decades. This will face the healthcare systems with new challenges. To find ways that optimize the utilization of the limited resources, it is important to understand which factors influence the outcomes at different points along the treatment pathway.

**Questions/purposes:**

We aimed to identify variables associated with physical function from hospital admission to discharge and at 12 months postsurgery (12 M). This study investigated for patients undergoing THA or TKA: What is the association between patients’ characteristics, surgery variables, and treatment decisions with patient-reported outcomes (PROs) at discharge as well as at 12 M?

**Patients/methods:**

We conducted a secondary, retrospective cohort analysis using longitudinal data from 6,144 THA and TKA patients who participated in the “PROMoting Quality Trial”. Physical function was assessed via the Hip Disability and Osteoarthritis Outcome (HOOS-PS) and Knee Injury and Osteoarthritis Outcome (KOOS-PS) scores. Stepwise selection and multivariate linear regression models were applied to identify variables associated with physical function at discharge and 12 M. The factors analyzed included surgery variables (surgeon presence, surgeon experience, surgery duration, complication) and treatment decisions (early mobilization, remote monitoring), along with patient characteristics.

**Results:**

We included 3,375 THA patients and 2,769 TKA patients. Admission HOOS-PS score, sex (being male), and early mobilization were the strongest predictors of better physical function at discharge for patients in the THA group, whereas admission HOOS-PS score, senior staff presence, and remote monitoring (intervention group) were significant predictors of better physical function for the THA patients at 12 M. For the patients in the TKA group, admission KOOS-PS score, early mobilization, and high surgeon experience were the strongest predictors of improved physical function at discharge. The admission KOOS-PS score, surgery duration, and being in the remote monitoring group were the strongest predictors of better physical function at 12 M.

**Conclusion:**

Early mobilization was significantly associated with better physical function at discharge from the clinic in both procedures, TKA and THA. The preoperative physical function scores and being allocated to the remote monitoring group were the strongest predictors of better physical function at 12 M.

**Supplementary Information:**

The online version contains supplementary material available at 10.1186/s42836-025-00313-2.

## Introduction

Care quality assessment and outcome transparency are gaining importance in the context of health care resource burden, patient centredness, and a shift toward value-based health care [1, 2]. To date, quality of care has been particularly measured with clinical outcomes in Germany [3] and other healthcare systems [4, 5]. However, patient-reported outcomes (PROs) measured by patient-reported outcome measures (PROMs) are becoming increasingly relevant internationally [3, 6]. PROs are health outcomes that reflect a change in health status as perceived by the patient and include outcomes such as symptom burden, functional status, or health-related quality of life (HrQoL) [7]. PROMs measure PROs from the patient’s perspective and can be used to assess treatment effectiveness [8, 9]. In the context of orthopedic surgeries, such as total hip arthroplasty (THA) and total knee arthroplasty (TKA), PROMs have been widely used in research [10], and some countries, such as Sweden, already collect PROMs routinely before and after arthroplasty surgery as part of registries [11]. By integrating PROs into routine clinical practice and outcome assessment, it is possible to gain a comprehensive understanding of patients’ experiences, preferences, and outcomes [12]. A patient-centered approach not only enhances transparency and accountability within healthcare systems but can also foster collaborative decision-making [13]. Continuous quality improvement initiatives [14] and improved resource allocation [15].

Although THA and TKA are highly standardized elective surgery procedures, high-quality variation between providers has been reported in the past [2, 16]. The measurement of care quality is a complex construct with multiple input factors potentially associated with quality variation. These factors include technical factors, such as implant type or surgical technique [17], hospital characteristics, such as hospital processes or quality management, and surgeon experience [18–21]. The literature has investigated the associations between patient characteristics and PROs quite thoroughly [22–25]. Additionally, some studies suggest that preoperative patient education programs [26], surgeon experience [24], and surgical technique [27–30] or other treatment decisions [31–33] are important factors influencing PRO variation. With respect to THA and TKA, PROs are usually collected at different intervals along the patient pathway, starting preoperatively and ending with postoperative assessments one or two years after surgery [10]. This can lead to varying sensitivity and specificity of PROs, depending on the points in time at which the measurements took place [34].

The influence of patient characteristics on PROs following THA and TKA at different time points has been extensively studied [35, 36]. However, understanding how certain treatment choices are associated with PROs at different time points is critical to optimizing the quality and effectiveness of orthopedic treatments such as THA and TKA. There is a notable gap in comprehensive studies that integrate clinical data with PROs to provide a holistic understanding of treatment effectiveness, not only on the basis of clinical outcomes but also from the patient’s perspective at different time points along the treatment pathway [6]. To the best of our knowledge, no previous study has investigated multiple factors influencing physical function based on data from a large clinical trial. This work aims to bridge this gap by investigating the associations between patient characteristics, surgery variables, and treatment decisions with physical function at discharge as well as at 12 months postsurgery (12 M) for patients receiving THA and TKA.

## Methods

### Study design

The present study is a retrospective cohort study that uses the data collected during the PROMoting Quality study. The reporting of this study followed the Strengthening the Reporting of Observational Studies in Epidemiology (STROBE) guidelines [37]. The PROMoting Quality study was a randomized clinical trial (RCT) with a parallel group design in which nine German hospitals participated from October 2019 to the end of December 2020. PROM-based monitoring, as a digital remote monitoring intervention, was tested in patients who underwent THA or TKA. Both THA and TKA patients were randomly assigned to a control or an intervention group (1:1 ratio) and remained blinded for the entire duration of the study. The clinical staff was blinded until the patients were discharged from the hospital. The RCT was conducted in accordance with a previously published study protocol [38].

This present cohort study uses a priori-specified secondary outcomes on patients’ physical function reflected by validated disease-specific PROM sets: the Hip Disability and Osteoarthritis Outcome Score-Physical Function Short Form (HOOS-PS) [39] and the Knee Injury and Osteoarthritis Outcome Score-Physical Function Short Form (KOOS-PS) [40]. The PROMoting Quality study was approved by the ethics committee of Charité Universitätsmedizin, Berlin (EA4/169/19) and was registered in the German Clinical Trials Register under trial number DKRS00019916.

### Setting/Intervention

All participants were digitally surveyed with validated, self-assessed PROMs at hospital admission, discharge, and 12 M. Physical function was assessed by validated disease-specific PROM sets. The intervention group received additional PROM assessments at one, three, and six months after discharge. Furthermore, automated digital alerts notified study nurses when predefined PRO thresholds were exceeded or if a 10% relative deterioration in the patient’s individual score was observed (remote monitoring intervention). The study nurses then contacted and informed patients, and, upon the patients’ agreement, the patients’ aftercare physicians were also informed about the alert and PROM results. With the use of PROM-based remote monitoring in the intervention group, individualized treatment path adjustments were possible whenever alerts were observed. Further details on the remote monitoring intervention are described in the PROMoting Quality study protocol [38].

### Participants

Initially, a total of 7,827 patients signed the consent form to participate in the PROMoting Quality study. Eligible patients who received THA or TKA were identified by appropriate surgery codes and were at least 18 years old. Participants were excluded from the study if they incorrectly received the remote monitoring (*n* = 59), planned surgery was not received (*n* = 564), discharge information was missing and therefore randomization was not possible (*n* = 326), or procedure codes did not match the prespecification (*n* = 71).

**Fig.1:**
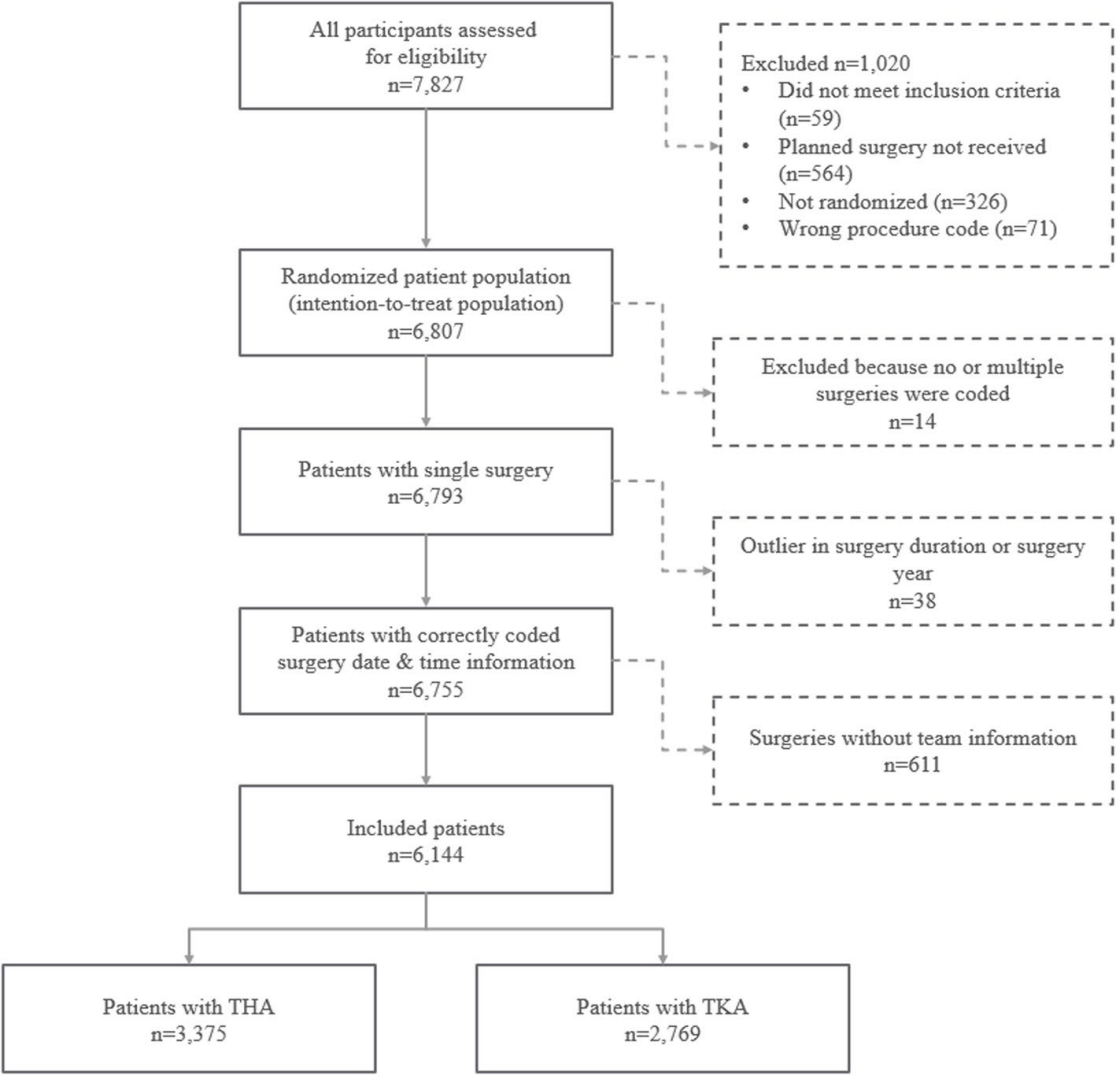
STROBE flowchart of patients considered in this study

After the removal of patients with no or multiple coded surgeries (*n* = 14), outliers in surgery duration or coded surgery date (*n* = 38) and patients without information on surgery team composition (*n* = 611), 6,144 patients with full data were included and retrospectively reviewed in this secondary analysis, with 3,375 patients having undergone THA and 2,769 TKA (STROBE flowchart in Fig. 1).

### Variables

This study investigated physical function changes at discharge and 12 M as outcomes, where discharge reflects the period between admission and discharge, and 12 M reflects the period between admission and 12 months postsurgery, and changes were measured by the PROM scores at admission and at the time of interest. Explanatory variables potentially include patient characteristics, surgery variables, and postoperative treatment decisions. Patient characteristics included the continuous variable of age, and the categorical variables of sex, body mass index group (BMI, assigned to groups underweight, normal, overweight, and obese on the basis of BMI results), education (higher vs lower, where higher means at least secondary degree), job status (able to work vs not able to work, where inability to work is caused by disease), living situation (whether living alone or with others), smoking status at admission, the sum of comorbidities (where collected comorbidities included cardiovascular, blood, lung, kidney, liver, cancer, depression, back-pain, arthritis, diabetes, and stroke), care grade presence at admission (care grade present when care grade > 0, German classification [41]), and surgery history (previous surgery present when joint-related surgical history present). Surgery variables included the presence of senior staff (measured in headcount; senior staff presence defined as headcount of chief or senior physicians ≥ 1), surgery duration in minutes, complications (yes vs no; a detailed description of assessed complications can be found in Supplementary Information File 1) and main surgeon experience (experienced vs not experienced, where experienced surgeons have performed > 150 surgeries of the respective procedure in the previous year; a detailed overview of procedure codes is provided in the Supplementary Information File 2). Postoperative treatment decisions included mobilization (where early mobilization was predefined as mobilization within six hours after surgery vs normal mobilization) and remote monitoring (yes vs. no, where yes reflects belonging to the intervention group). A detailed description of the variables, including specifications, can be found in Supplementary Information File 1.

### Statistical analysis

This study followed a two-step statistical approach. First, to identify the significance of the relevant parameters to be included in the model estimation, separate step-forward selections on discharge and 12 M periods were conducted. Second, multiple linear regression models were used to estimate the associations between the variables of interest and improvements in physical function at discharge and 12 M in patients in the THA/TKA patient groups. Missing data were omitted from each regression.

In the step-forward selection, variables were included stepwise to an empty model until further inclusion of variables could not add any significant contribution to the performance of the regression model, on the basis of the decrease in the model Akaike information criterion (AIC, significant when the AIC decreased ≥ 2 [42]). Variable importance plots were obtained for each outcome and survey period with full data, ranking potentially associated variables by the decrease in the AIC upon their inclusion (step forward) or increase upon their removal (step backward). This method has already been used in similar health care research [24, 43, 44]. The advantages of step-forward selection are that it starts with smaller models and is less susceptible to collinearity. On the other hand, in forward selection, a new variable may result in the nonsignificance of other already included variables that cannot be deleted from the model [45]. Therefore, the sensitivity of selection was checked by running separate step-backward selections and comparing variable inclusion. Additionally, we ran a bootstrap algorithm to validate each regression model with 1,000 bootstrap samples to understand the likely variability of association factors within the study population [46, 47]. After the identification of relevant variables, separate multiple regression models were developed for THA and TKA for both time intervals.

All data management and data analyses were performed via R v. 2024.04.2 build 764. All significance tests were 2-sided with an alpha level of 0.05. Categorial variables are presented as patient volumes and percentages of the overall study population. Continuous variables are presented as the means and standard deviations.

## Results

### Descriptives

A summary of the descriptive characteristics of the study population can be found in Table 1. A total of 6,144 patients were included in this study; 3,375 patients received THA, and 2,769 patients received TKA. Within the study, 44.9% of the patients were men (THA: 44.2%; TKA: 45.8%), and the mean age of the participants was 70.3 years (THA: 70.2 ± 10.6; TKA: 70.4 ± 9.2). The mean HOOS-PS and mean KOOS-PS at admission were 47.7 ± 16.3 and 43.1 ± 12.7, respectively. Most patients who completed the 12 M survey experienced an improvement in physical function in PROs (THA: 96.7%; TKA: 88.0%). The mean improvement in physical function for patients in the THA group was 1.3 at discharge and 33.0 at 12 M. In contrast, patients in the TKA group experienced a mean decline in physical function of 1.6 at discharge, followed by a mean improvement of 17.1 at 12 M. The loss to follow-up ratios were 17.4% (587) and 19.8% (549) in the THA and TKA patient groups, respectively.

**Table 1:** Descriptive characteristics of THA and TKA patients at admission

Variable	Patients in THA group	Patients in TKA group
Total, *n*	3,375	2,769
Patient characteristics		
Mean age, yrs (SD)	70.2 (10.6)	70.4 (9.2)
Mean BMI, kg/m^2^ (SD)	28.0 (5.3)	30.5 (5.8)
Underweight (BMI ≤ 18.5), *n* (%)	19 (0.6%)	4 (0.1%)
Normal (18.5 < BMI ≤ 25), *n* (%)	1,058 (31.4%)	415 (15.0%)
Overweight (25 < BMI ≤ 30), *n* (%)	1,287 (38.2%)	1,047 (37.8%)
Obese (BMI > 30), *n* (%)	1,006 (29.9%)	1,303 (47.1%)
Sex (M:F), *n* (%)	1,492:1,883 (44.2%:55.8%)	1,269:1,500 (45.8%:54.2%)
Living situation (alone:with others), *n* (%)	781:2,590 (23.2%:76:8%)	534:2,235 (19.3%:80.7%)
Job (able to work:not able), *n* (%)	3,037:334 (90.1%:9.9%)	2,442:327 (88.2%:11.8%)
Smoker at adm (yes:no), *n* (%)	520:2,851 (15.4%:84.6%)	359:2,410 (13.0%:87.0%)
Mean sum of comorbidities (SD)	1.6 (1.0)	1.7 (1.0)
Care grade at adm (y:n), *n* (%)	100:3,271 (3.0%:97.0%)	77:2,692 (2.8%:97.2%)
Previous surgery (y:n), *n* (%)	1,631:1,744 (48.3%:51.7%)	2,029:740 (73.3%:26.7%)
Education (higher:lower), *n* (%)	2,927:444 (86.8%:13.2%)	2,296:473 (82.9%:17.1%)
Surgery variables		
Surgeon exp. (reported:n/a), *n* (%)	2,826:549 (83.7%:16.7%)	2,229:540 (80.5%:19.5%)
Not experienced, *n* (% of reported)	1,104 (39.1% of reported)	823 (36.9% of reported)
Experienced, *n* (% of reported)	1,722 (60.9% of reported)	1,406 (63.1% of reported)
Senior staff present (y:n), *n* (%)	3,120:255 (92.4%:7.6%)	2,472:297 (89.3%:10.7%)
Surgery duration, minutes (SD)	64.4 (23.7)	71.5 (22.4)
Complication (y:n), *n* (%)	82:3,293 (2.4%:97.6%)	22:2,747 (0.8%:99.2%)
Treatment decisions		
Mobilization (early:normal), *n* (%)	1,596:1,775 (47.4%:52.6%)	1,314:1,455 (47.5%:52.4%)
Remote monitoring (y:n), *n* (%)	1,692:1,683 (50.1%:49.9%)	1,393:1,376 (50.3%:49.7%)
PROMs (HOOS-PS/KOOS-PS)		
Mean admission PROM score (SD)	47.7 (16.3)	43.1 (12.7)
Mean discharge PROM score (SD)	46.4 (17.6)	44.7 (12.1)
Mean 12 M PROM score (SD)	14.7 (14.5)	26.0 (14.2)

BMI = body mass index. HOOS-PS and KOOS-PS measure physical functioning in patients with THA and TKA, where a lower PROM score signals higher physical functioning

### Variable selection—analysis of variable inclusion in model estimation

#### Association of surgery variables and treatment decisions with physical function outcomes in the THA group

**Fig. 2:**
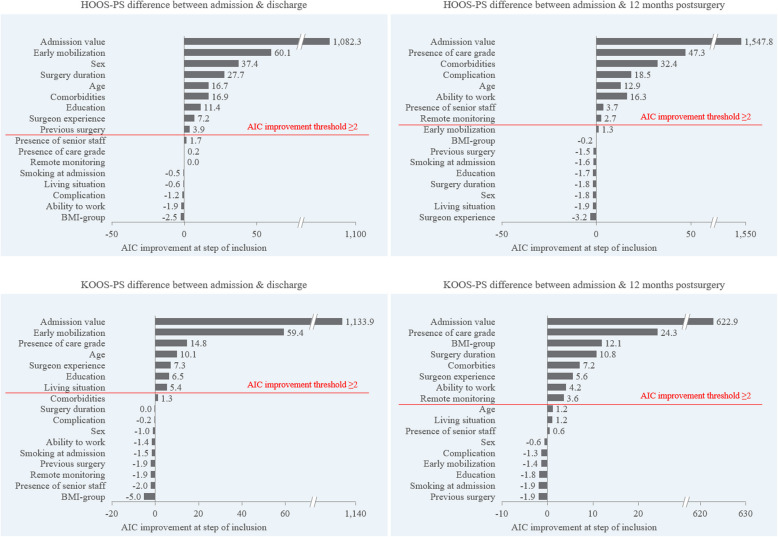
Variable importance plots are displayed for HOOS-PS and KOOS-PS differences at discharge and 12 M, which are based on a decrease in the AIC when the variables are included

The step-forward selection suggested that nine variables be included for linear regression of differences in the HOOS-PS score at discharge, whereas eight variables should be included for the 12 M time frame. The baseline HOOS-PS score was the variable associated with the greatest improvement in the physical function of the patients in the THA patient group at discharge and 12 M (AIC decrease: 1,082.3 and 1,547.8, respectively). See Fig. 2 for a detailed assessment of the variance importance factor of each tested variable.

##### At discharge

Early mobilization was the second most important variable for changes in physical function at discharge (AIC decrease: 60.1 for HOOS-PS). Additional significant contributors and antagonists to physical function at discharge included sex (AIC decrease: 37.4), surgery duration (AIC decrease: 27.7), age (AIC decrease: 16.7), comorbidities (AIC decrease: 16.9), education (AIC decrease: 11.4), surgeon experience (AIC decrease: 7.2), and previous surgery (AIC decrease: 3.9).

##### At 12 months post-surgery

At 12 M, care grade had the second highest association with changes in physical function (AIC decrease: 47.3). Additional contributors and antagonists to changes in physical function were comorbidities (AIC decrease: 32.4), complications (AIC decrease: 18.5), age (AIC decrease: 12.9), ability to work (AIC decrease: 16.3), presence of senior staff (AIC decrease: 3.7), and remote monitoring (AIC decrease: 2.7).

#### Association of surgery variables and treatment decisions with physical function outcomes in the TKA group

For the TKA group and the respective model with the delta in the KOOS-PS outcomes as the dependent variable, the step-forward selection suggested seven variables to be included at discharge, whereas eight variables should be included for the 12 M timeframe. Similar to THA, for TKA patients, the baseline KOOS-PS score exhibited the strongest association with changes in physical function both at discharge and at 12 M (AIC decrease: 1,133.9 and 622.9, respectively). Figure 2 shows the detailed assessment of the variance importance factor of each tested variable.

##### At discharge

Early mobilization was the second most important variable for changes in physical function at discharge (AIC decrease: 59.4). Surgeon experience (AIC decrease: 7.3) was less important than care grade (AIC decrease: 14.8) and age (AIC decrease: 10.1), yet it ranked higher than education and living situation (AIC decrease: 6.5 and 5.4, respectively).

##### At 12 months postsurgery

For 12 M, care grade had the second highest association with changes in physical function (AIC decrease: 24.3). Surgeon experience was less important (AIC decrease: 5.6) than care grade (AIC decrease: 24.3), BMI group (AIC decrease: 12.1), surgery duration (AIC decrease: 10.8), and comorbidies (AIC decrease: 7.2); however, it had a stronger effect on physical function than ability to work or the remote monitoring (AIC decrease: 4.2 and 3.6, respectively).

### Sensitivity analysis for variable selection analysis

Confirming the robustness of the variable selection, step-backward selection suggested removing the same variables that were not to be included in the step-forward selection for both PROMs at discharge and 12 M. Bootstrap testing revealed significant confidence intervals for all selected variables for THA and TKA patients at both time intervals.

### How do the selected variables influence physical function?

#### Influence of selected variables on physical function in THA patients

A model summary of THA physical function is presented in Table 2. The HOOS-PS admission value was significantly associated with improvements in physical function (β = 0.575 at discharge and β = 0.690 for 12 M, *P* < 0.001 each). Similarly, age (β = 0.074 (discharge)/β = − 0.075 (12 M), *P* < 0.001) and the sum of comorbidities (β = − 0.061 (discharge)/β = − 0.066 (12 M), *P* < 0.001) had significant effects on physical function changes in both models. The variables with a significantly positive impact on physical function improvement at discharge were male sex (β = 0.097, *P* < 0.001), early mobilization (β = 0.096, *P* < 0.001), higher education (β = 0.057, *P* < 0.001), and high surgeon experience (β = 0.034, *P* = 0.042), whereas surgery duration and present surgery history were significantly associated with a decrease in physical function (β = − 0.065, *P* < 0.001 and β = − 0.035, *P* = 0.018). At 12 M, being in the remote monitoring group (β = 0.030, *P* = 0.032) and senior staff presence (β = 0.036, *P* = 0.018) were associated with better physical function. On the other hand, care grade (β = − 0.090), 12 M inability to work (β = − 0.064), and complications (β = − 0.060) significantly (*P* < 0.001) led to lower improvements in physical function in THA patients at 12 M.

**Table 2:** Multiple linear regression of differences in the HOOS-PS scores for THA patients at discharge and 12 M

	**(1) Difference in HOOS-PS (discharge)**						**(2) Difference in HOOS-PS (12 M)**				
	**B**	**SE**	**ß**	**t**	***P***	**B**		**SE**	**ß**	**t**	***P***
Intercept	− 40.066	2.406	− 0.005	− 16.649	< 0.001	5.056		2.160	0.002	2.341	0.019
HOOS-PS admission value	0.690	0.018	0.575	38.012	< 0.001	0.776		0.016	0.690	47.468	< 0.001
Patient characteristics											
(D) Sex – male	3.803	0.580	0.097	6.562	< 0.001						
Age	0.137	0.028	0.074	4.928	< 0.001	− 0.130		0.026	− 0.075	− 4.954	< 0.001
(D) Ability to work – no						− 3.896		0.916	− 0.064	− 4.252	< 0.001
Sum of comorbidities	− 1.254	0.311	− 0.061	− 4.028	< 0.001	− 1.275		0.291	− 0.066	− 4.375	< 0.001
(D) Care grade presence – yes						− 9.753		1.697	− 0.090	− 5.748	< 0.001
(D) Surgery history – yes	− 1.387	0.573	− 0.035	− 2.419	0.016						
(D) Education – higher	3.314	0.843	0.057	3.931	< 0.001						
Surgery variables											
(D) Main surgeon experience – experienced	1.322	0.651	0.034	2.030	0.042						
(D) Main surgeon experience – not reported	2.563	0.875	0.048	2.927	0.003						
(D) Presence of senior staff – yes						2.469		1.043	0.036	2.367	0.018
Surgery duration	− 0.054	0.013	− 0.065	− 4.300	< 0.001						
(D) Complication – yes						− 7.095		1.654	− 0.060	− 4.290	< 0.001
Treatment decisions											
(D) Mobilization – early	3.765	0.578	0.096	6.510	< 0.001						
(D) Remote monitoring – yes						1.099		0.512	0.030	2.146	0.032

Model (1) Observations = 3,269; R^2^ = 0.325; Adj. R^2^ = 0.323; F(10,3258) = 156.7; *P* < 0.001

Model (2) Observations = 2,829; R^2^ = 0.451; Adj. R^2^ = 0.450; F(8,2820) = 290.1; *P* < 0.001

(D) = Dichotomous variable. Also see Additional File 1 for further information on variables

### Influence of selected variables on physical function in TKA patients

A model summary for TKA physical function can be found in Table 3. Only the KOOS-PS admission value (β = 0.610 (discharge)/β = 0.522 (12 M)) and care grade (β = − 0.067 (discharge)/β = − 0.096 (12 M)) were significantly (*P* < 0.001) associated with improvements in physical function at both time intervals. At discharge, early mobilization (β = 0.113, *P* < 0.001), high surgeon experience (β = 0.056, *P* = 0.002), age (β = 0.053, *P* < 0.001), and higher education (β = 0.045, *P* < 0.001) were significantly associated with improvements in physical function. TKA patients living alone had significantly (*P* = 0.006) lower improvements at discharge (β = − 0.042). For 12 M, surgery duration (β = 0.058, *P* = 0.002) and being in the remote monitoring group (β = 0.042, *P* = 0.018) were associated with better physical function. In contrast, an obese BMI (β = − 0.073, *P* = 0.001, a higher number of comorbidies (β = − 0.059, *P* = 0.001), and inability to work (β = − 0.046, *P* = 0.013) were associated with lower improvements in physicial function.

**Table 3:** Multiple linear regression of differences in the KOOS-PS scores for TKA patients at discharge and 12 M

	**(1) Difference in KOOS-PS (discharge)**						**(2) Difference in KOOS-PS (12 M)**				
	**B**	**SE**	**ß**	**t**	***P***	**B**		**SE**	**ß**	**t**	***P***
Intercept	− 37.974	1.917	− 0.001	− 19.809	< 0.001	− 8.615		1.548	− 0.008	− 5.564	< 0.001
KOOS-PS admission value	0.660	0.017	0.610	38.550	< 0.001	0.612		0.022	0.522	27.818	< 0.001
Patient characteristics											
Age	0.079	0.023	0.053	3.396	< 0.001						
(D) BMI group – underweight						0.460		6.454	0.001	0.071	0.943
(D) BMI group – overweight						− 0.227		0.817	− 0.007	− 0.278	0.781
(D) BMI group – obese						− 2.187		0.812	− 0.073	− 2.694	0.007
(D) Living situation – alone	− 1.460	0.536	− 0.042	− 2.725	0.006						
(D) Ability to work – no						− 2.109		0.853	− 0.046	− 2.473	0.013
Sum of comorbidities						− 0.925		0.291	− 0.059	− 3.183	0.001
(D) Care grade presence – yes	− 5.605	1.310	− 0.067	− 4.278	< 0.001	− 8.694		1.809	− 0.096	− 4.806	< 0.001
(D) Education – higher	1.644	0.567	0.045	2.901	0.004						
Surgery variables											
(D) Main surgeon experience – experienced	1.546	0.487	0.056	3.176	0.002	− 0.970		0.625	− 0.033	− 1.552	0.121
(D) Main surgeon experience – not reported	1.441	0.624	0.042	2.310	0.021	− 2.511		0.843	− 0.067	− 2.978	0.003
Surgery duration						0.038		0.012	0.058	3.161	0.002
Treatment decisions											
(D) Mobilization – early	3.120	0.426	0.113	7.324	< 0.001						
(D) Remote monitoring – yes						1.268		0.536	0.042	2.367	0.018

Model (3) Observations = 2,725; R^2^ = 0.369; Adj. R^2^ = 0.367; F(8,2716) = 198.3; *P* < 0.001

Model (4) Observations = 2,302; R^2^ = 0.266; Adj. R^2^ = 0.263; F(11,2290) = 75.6; *P* < 0.001

(D) = Dichotomous variable. Also see Additional File 1 for further information on variables

## Discussion

This study revealed that both surgery variables and post-operative treatment decisions, as well as selected patient characteristics, were associated with improvements in physical function in patients receiving THA or TKA. Stepwise variable selection was used for simplified parameter selection and resulted in accurate multiple linear regression models validated by the bootstrap algorithm.

Early mobilization, as part of the treatment decision, significantly increased physical function in both the THA and TKA patient groups, at least at discharge. Treatment with PROM-based remote monitoring was associated with improvements in physical function at 12 M. These findings align with those of previous studies that reported fewer postoperative complications and increased patient satisfaction following early mobilization, suggesting that early mobilization is a safe and effective post-operative treatment [48]. Additionally, earlier studies revealed similar effects of PROM-based remote monitoring on overall HrQoL and fatigue and on physical functioning at 12 M [49].

Furthermore, findings from another study explored existing intersurgeon variability that could not be explained by the surgical approach and suggested incorporating surgeon experience as a variable within model fitting [24]. Our findings support the importance of surgeon experience for physical function at discharge rather than at 12 M, which is consistent with the findings of Sinclair et al., although their study revealed a broader range of surgeon experience (4–764 THAs per surgeon) [24]. Considering complications, our study supports findings from previous research that investigated adverse events in THA and TKA, revealing significantly lower improvements in physical function for THA patients at 12 months postsurgery, while no differences were observed at discharge [50]. Our study could also identify significant effects in THA only at 12 M and no significance for TKA in both time intervals, possibly due to low overall complication rates, which can be attributed to the high-volume surgical centers contributing data to the PROMoting Quality study, and this secondary analysis [51].

Previous studies have indicated that older age groups tend to have lower overall PROM scores and less significant improvement in PROs at 12–24 months postsurgery [52, 53]. This study revealed similar effects in the long term, but revealed that improvement at discharge was greater in older patients. The difference could be explained by the measured timeframes: the mentioned publications focused on outcomes 12–24 months after surgery, while this study specifically also examined the association at discharge with improvements in physical function. Our findings of higher improvements in physical function at discharge with increasing age are consistent with a recent systematic review reporting mixed results, with some studies reporting higher PROM outcomes in older patients, while others reported the opposite [54]. This highlights the importance of differentiating between discharge and 12 M associations in terms of physical function. Comorbidities were significantly associated with lower improvements in physical function in the THA patient group at both discharge and 12 M. Braaksma et al. reported a small predictive performance of patient characteristics on the HOOS-PS [55], which aligns with our findings of significantly lower, yet small, improvements in physical function. Additionally, patients with care grade (i.e., those with long-term care needs) achieved significantly lower improvements in physical function at 12 M, which is consistent with the findings of previous studies, which identified difficulties in assimilating information and rehabilitation at home [56].

Our study features several important strengths. The robustness of this study was enhanced by the RCT design of the PROMoting Quality trial with strong internal validity and a large-scale dataset at the patient level from nine different German hospitals. This study investigated associations with physical function after THA or TKA and differentiated between discharge and 12 M. While THA and TKA are fundamentally different procedures in terms of surgical approach and post-operative treatment, they are both highly standardized elective orthopedic procedures. Postoperatively, patients undergoing TKA tend to experience less improvement than those undergoing THA [57]. Our large dataset enabled separate analysis, allowing for clinically meaningful insights tailored to each procedure. Another major strength of the study is the methodolical robustness. The study used a two-step approach in variable selection, and sensitivity was checked by using bootstrap algorithms.

However, certain limitations should be noted. This study predominantly included patients from high-volume surgical centers, limiting the generalizability of the findings to all German hospitals. Furthermore, the latest follow-up PROM collection was restricted to 12 M, precluding conclusions about longer-term outcomes and associated variables. Additionally, further surgeries, even if unrelated to the investigated joints, may have impacted physical function recovery but were not assessed in this study. Finally, data for some variables, such as surgery team composition, were missing for certain surgeries, preventing conclusions from being drawn for PROs in these patients.

## Conclusions

Given the growing importance of quality of care as assessed by PROMs in patient treatment, it is crucial to identify the specific parameters that are significantly associated with the improvement of PROs and how these associations might differ over time. By testing variables associated with physical function, this study revealed that early mobilization is significantly associated with improvements in the physical function of THA and TKA patients. Specifically, after physical function admission values, early mobilization was the second-highest associated variable for improvement in physical function at discharge for both patient groups. Therefore, healthcare providers should prioritize the revision of patient care pathways to incorporate early mobilization protocols for all patients undergoing THA and TKA. Patient characteristics, such as care grade, comorbidities, and BMI group, are becoming increasingly associated with physical function recovery over time, whereas the influence of treatment decisions on physical function decreases over time. Complications had a significant impact on the improvement of physical function in THA and should be prevented or minimized to optimize patient outcomes. Future research should explore association variance in an even longer time frame and allow further adaptation of patient treatment on the basis of desired outcome improvement and timeline.

## Supplementary Information

Additional file 1. Detailed variable description.

Additional file 2. Table S1. Procedure Codes (OPS) for inclusion of patients.

## Data Availability

The datasets generated and analyzed in the current study, as well as the full collected study dataset, are not publicly available due to data protection regulations. Access to data is limited to the researchers who have obtained permission for data processing. Further inquiries can be made to the corresponding author.

## Abbreviations

12 M: 12 months postsurgery
AIC: Akaike information criteria
BMI: Body mass index
HOOS-PS: Hip Disability and Osteoarthritis Outcome Score-Physical function short form
HrQoL: Health-related quality of life
KOOS-PS: Knee Injury and Osteoarthritis Outcome Score-Physical function short form
PRO: Patient-reported outcome
PROM: Patient-reported outcome measure
RCT: Randomized clinical trial
THA: Total hip arthroplasty
TKA: Total knee arthroplasty
