# Supplementary material for: Treatment decisions and surgery variables are predictors of better physical function after total hip and knee arthroplasty: a retrospective cohort study
Source: Arthroplasty. 2025 Jun 4;7:29. doi: 10.1186/s42836-025-00313-2 (PMC12135223; doi:10.1186/s42836-025-00313-2)
Supplement: Supplementary file 1 — Additional file 1. Detailed variable description. [file 42836_2025_313_MOESM1_ESM.pdf]

Cluster/Variable	Specification	Explanation
**Patient characteristics**		
Age	*0-∞ years*	Continuous variable
BMI group	*Underweight/Normal/Overweight/Obese*	Underweight = BMI<18.5; Normal = 18.5<BMI<25; Overweight = 25<BMI<30; Obese = BMI >30
Education	*High vs low*	Dichotomous variable; higher education = secondary or university degree; lower education = no or primary degree
Sex	*Male vs female*	Dichotomous variable; patient gender
Job status	*Able to work vs not able to work*	Dichotomous variable
Living situation	*Living alone vs living with others*	Dichotomous variable; living alone if no other persons is living with the patient
Smoking status	*Smoker vs non-smoker*	Dichotomous variable
Sum of comorbidities	*0-∞ comorbidities*	Index variable; Sum of cardio, blood, circulation, lung, kidney, liver, cancer, depression, back-pain, arthritis, diabetes, and stroke comorbidities
Care grade presence	*Yes vs no*	Dichotomous variable; care grade present if care grade at admission >0
Surgery history	*Yes vs no*	Dichotomous variable; surgery history present for joint-related surgical history
**Intraoperative variables**		
Presence of senior staff	*Yes vs no*	Dichotomous variable; Senior staff = chief or senior physician
Surgery duration	*10-1,440 minutes*	Continuous variable; measured in minutes; excluded surgeries with unrealistic surgery duration
Complication	*Yes vs no*	Dichotomous variable; Complication = yes, when general or treatment-specific complication occurred<br>Measured general complications: pneumonia, treatment-requiring cardiovascular complication, deep leg/pelvic vein thrombosis, pulmonary embolism, catheter-associated urinary tract infection, stroke, acute gastrointestinal bleeding, renal insufficiency, other general treatment-requiring complication,<br>Measured THA-specific complications: primary implant malposition, secondary implant dislocation, open and closed reduced endoprosthesis (sub)luxation, surgery and intervention-requiring bleeding/hematoma, surgery or intervention-requiring vascular lesion, preoperative-requiring secondary necrosis of wound edges, other specific treatment-requiring complication<br>Measured TKA-specific complications: primary implant malposition, secondary implant dislocation, post-operative dislocation of artificial joint, surgery and intervention-requiring bleeding /hematoma, patella malalignment, persistent motor nerve damage, periprosthetic fracture, reoperation-requiring secondary necrosis of wound edges, postoperative mechanical complication of the artificial joint, rupture of the quadriceps tendon/patellar ligament, patella fracture, other specific treatment-requiring complications
Main surgeon experience	*Experienced vs not experienced*	Dichotomous variable; experienced = main surgeon conducting >=150 surgeries in previous year in respective procedure
**Postoperative variables**		
Physical function	*0-25 score*	Continuous variable; Measured by HOOS-PS & KOOS-PS, where a lower score signals higher physical functioning
Mobilization	*Normal vs early*	Dichotomous variable; early mobilization = mobilization within 6 hours
Remote monitoring	*Intervention vs control group*	Dichotomous variable; Intervention group received more PROM surveys and critical alerts
