# Supplementary material for: Treatment decisions and surgery variables are predictors of better physical function after total hip and knee arthroplasty: a retrospective cohort study
Source: Arthroplasty. 2025 Jun 4;7:29. doi: 10.1186/s42836-025-00313-2 (PMC12135223; doi:10.1186/s42836-025-00313-2)
Supplement: Supplementary file 2 — Additional file 2. Table S1. Procedure Codes (OPS) for inclusion of patients. [file 42836_2025_313_MOESM2_ESM.docx]

| **Table S1.** Procedure Codes (OPS) for inclusion of patients   \| Procedure codes^a^ \| The following procedure codes were included:  THA: 5-820.00, 5-820.01, 5-820.02, 5-820.20, 5-820.22, 5-820.8, 5-820.80, 5-820.81, 5-820.82, 5-820.9, 5-820.92, 5-820.93, 5-820.94, 5-820.95, 5-820.96, 5-820.X, 5-820.x0, 5-820.x1, 5-820.x2, 5-820.y  TKA: 5-822.0, 5-822.00, 5-822.01, 5-822.02, 5-822.g, 5-822.g0, 5-822.g1, 5-822.g2, 5-822.j, 5-822.j1, 5-822.j2, 5-822.k, 5-822.k0, 5-822.k1, 5-822.k2, 5-822.h1, 5-822.h2 \| \| --- \| --- \| |
| --- | --- | --- |

^a^ The German procedure classification (Operationen- und Prozedurenschlüssel—OPS) is the official classification for the encoding of operations, procedures, and general medical measures.
